# N^6^-methyladenosine (m^6^A) methylation in ischemia–reperfusion injury

**DOI:** 10.1038/s41419-020-2686-7

**Published:** 2020-06-24

**Authors:** Weifeng Yao, Xue Han, Mian Ge, Chaojin Chen, Xue Xiao, Haobo Li, Ziqing Hei

**Affiliations:** 1 Department of Anesthesiology, The Third Affiliated Hospital of Sun Yat-sen University, Guangzhou, 510630 China; 2 Department of Anesthesiology, Sun Yat-sen Memorial Hospital, Sun Yat-sen University, Guangzhou, 510000 China; 3 Department of Corrigan-Minehan Heart Center and Cardiology Division, Massachusetts General Hospital, Harvard Medical School, Boston, MA 02114 USA

**Keywords:** Epigenetics, Post-translational modifications

## Abstract

Ischemia–reperfusion (I/R) injury is common during surgery and often results in organ dysfunction. The mechanisms of I/R injury are complex, diverse, and not well understood. RNA methylation is a novel epigenetic modification that is involved in the regulation of various biological processes, such as immunity, response to DNA damage, tumorigenesis, metastasis, stem cell renewal, fat differentiation, circadian rhythms, cell development and differentiation, and cell division. Research on RNA modifications, specifically N6-methyladenosine (m^6^A), have confirmed that they are involved in the regulation of organ I/R injury. In this review, we summarized current understanding of the regulatory roles and significance of m^6^A RNA methylation in I/R injury in different organs.

## Facts

- m^6^A modifications have revealed relatively complete enzymatic systems, and the related regulatory mechanisms are becoming increasingly well understood.
- m^6^A mRNA modifications have been found in myocardial, brain, and renal ischemia–reperfusion (I/R) in in vivo and in vitro studies.
- m^6^A RNA modifications have been identified as an important mechanism in I/R-induced organ injury, where inhibition of m^6^A methylation protects organs from I/R injury.
- Most of the known functions of m^6^A have been inferred from the phenotypic consequences of manipulating m^6^A “writers” and “erasers” during I/R.

## Questions

- Does m^6^A modification play a regulatory role in ischemia or in reperfusion stage?
- What are the individual mRNA m^6^A modification sites during I/R?
- Can m^6^A mRNA methylation optimize ischemic cardiac preconditioning or post-conditioning in I/R therapy?

## Introduction

Ischemia, occurs following restricted blood supply to tissues, is common in patients undergoing surgeries. Re-establishment (reperfusion) of blood flow is mandatory to salvage the ischemic tissues^1–4^. In most cases, post-ischemic reperfusion can restore the normal functions of tissues and organs and repair damaged structures, but in some cases, reperfusion itself may lead to further tissue damage/dysfunction and eventually cause organ failure. This phenomenon is known as ischemia–reperfusion (I/R) injury. Despite incredible advancements of techniques in reducing tissue ischemia such as thrombolytic therapy, percutaneous coronary angioplasty and cardiopulmonary bypass, post-operative morbidity and mortality owing to I/R injury remain high^1–4^. Extensive studies have focused on investigating the underline mechanisms of I/R injury. Different mechanisms have been suggested, such as burst of reactive oxygen species (ROS) during reperfusion^5–7^, elevation for inflammatory response, mitochondrial dysfunction, and calcium overload. However, the full picture of the pathophysiology of I/R injury is far from complete and further research is warranted.

Epigenetic modifications, including histone modification and DNA methylation, have been demonstrated to play key roles in I/R injury^8,9^. Chromatin structure depends on electrostatic interactions between positive charges on histones and negative charges on DNA. Histones acetylation on lysine residues can neutralize the positive charge, thereby disrupting the stability of histone and DNA interactions, and subsequently changes the condensed chromatin into an open, loosely assembled chromatin structure that allows for the mobilization of gene transcription regulators^10^. DNA methylation is catalyzed by DNA methyltransferase, which adds methyl groups to DNA nucleotides, leading to chromatin condensation and gene expression changes^11^. Similarly, RNA nucleotides can also have covalent modifications that regulate gene expression by affecting RNA stability and translation. RNA modifications are types of post-transcriptional regulation, and over 150 types of RNA modifications have been identified. They are widely distributed in various types of RNA, including messenger RNA (mRNA), transfer RNA (tRNA), ribosomal RNA (rRNA), small non-coding RNA, and long non-coding RNA (lncRNA). RNA methylation accounts for over 60% of all RNA modifications^12,13^. Methylation modifications have been identified on all four ribonucleotides (A, U, C, and G) including N6-methyladenosine (m^6^A), 5-methylcytosine (m^5^C)^14^, 3-methyluracil (m^3^U)^15^, N7-methylguanosine (m^7^G)^16^, and so on. Among them, m^6^A methylation is the most common type of RNA methylation in mRNA^17^. m^6^A methylation primarily occurs at the sixth site of adenine in RRACH RNA sequences, which can regulate the splicing, transportation, localization, translation, and degradation of target RNAs^18^. m^6^A is widely present in various eukaryotes, including yeast^19^, plants^20^, *Drosophila*^21^, and mammals^18^, and it is also the most common modification in lncRNA in higher organisms^22–24^. However, specific molecular functions of m^6^A are still not well understood.

In recent years, studies on m^6^A RNA modification have confirmed that it dynamically and reversibly regulates the development and progression of I/R injury in different organs. In this review, we summarized the known modifications, regulations, and significances of m^6^A RNA methylation in I/R injury in different organs.

## m^6^A RNA methylation machinery

m^6^A modifications on mRNA was first reported by Desrosiers et al.^18^ in the 1970s, but studies on RNA modification have lagged behind studies on DNA modifications, and precise functions of m^6^A modifications were largely unclear until recently. The core question that remains unresolved regarding RNA modifications is whether such modifications regulate gene expression and whether they are dynamic and reversible.

**Fig. 1:**
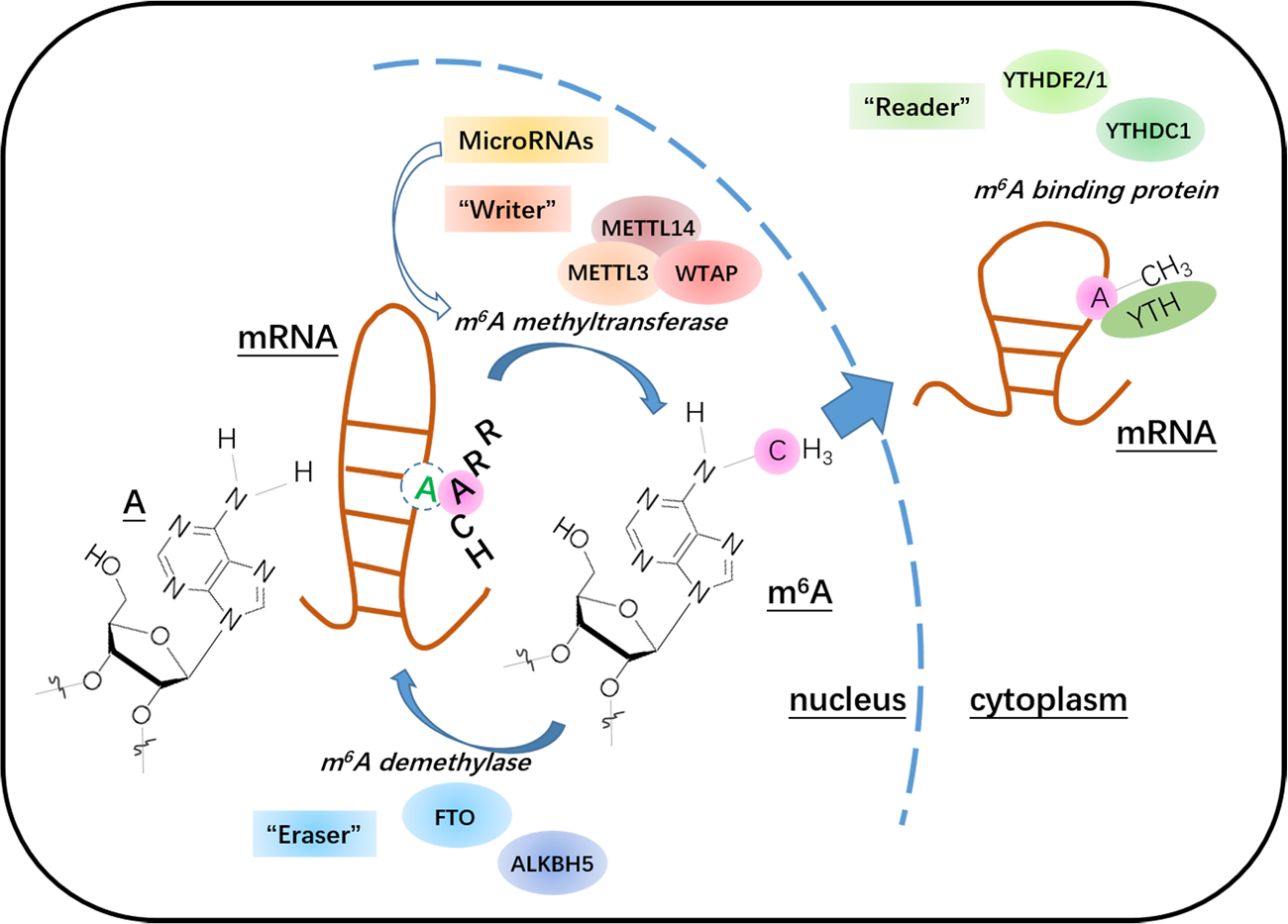
The dynamic and reversible process of m6A modification of RNA and its potential functions in the regulation of mRNA processing and metabolism. **a** m^6^A modification occurs primarily at sixth site of adenine in RRACH sequences (R indicates A or G; H indicates A, U, or C). Dynamic and reversible mRNA m^6^A modification: the m^6^A methyltransferase complex METTL3/METTL14/WTAP catalyzes the transformation of A to m^6^A, and the demethylases FTO and ALKBH5 catalyze its demethylation. The YTH family protein YTHDC1 binds to m^6^A modifications in the nucleus, and YTHDF2/1 binds to cytoplasmic m^6^A. MicroRNAs regulate m^6^A production by regulating METTL3 activity.

The functions of m^6^A methylation are determined by “writers”, “erasers”, and “readers”^25^ (Fig. 1). “Writers”, also known as methyltransferases, are proteins that induce specific RNA methylation. Methyltransferase-like protein 3 (METTL3), methyltransferase-like protein 14 (METTL14), Wilm’s tumor 1 associated protein (WTAP), KIAA1429, METTL3, METTL14, and WTAP are methyltransferases that catalyze m6A modifications^26^. METTL3 and its homolog METTL14 are localized in splicing factor-rich nuclear subcellular compartments known as nuclear speckles, suggesting that m^6^A modifications may be associated with RNA splicing. WTAP interacts with METTL3–METTL14 dimers and colocalizes with nuclear speckles, which affects the efficiency of methylation and mRNA splicing^27^. KIAA1429 is a candidate subunit of the methyltransferase complex that has been shown to be necessary for methylation^28^.

**Fig. 2:**
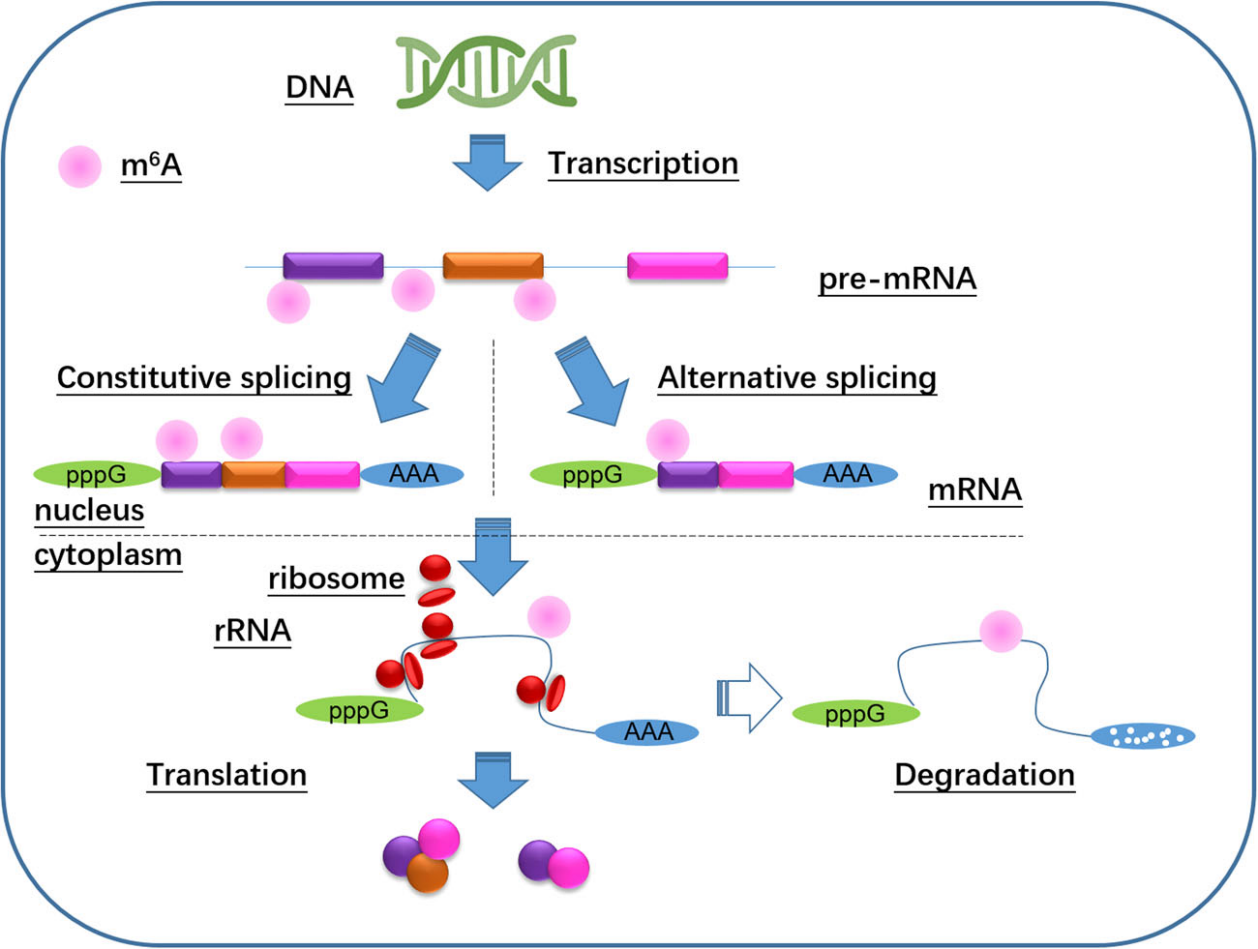
Methylation and demethylation of m6A mRNA modifications occur primarily in the nucleus. m^6^A also exists in the intronic regions of pre-mRNA precursors, suggesting that methylation modifications may regulate the alternative splicing of mRNA precursors to form mature mRNAs. m^6^A also affects the nuclear export, translation, and degradation of mRNA; mRNA degradation is mediated by YTHDF2, mRNA translation is mediated by YTHDF1, and alternative splicing is mediated by YTHDC1; other biological functions are yet to be elucidated. The potential regulatory functions of m^6^A in mRNA processing and metabolism, including splicing, nuclear export, localization, translation, and degradation (stability), occur after the transcription of precursor pre-mRNA, and m^6^A is an important reversible chemical modification of mRNA that may serve as a novel cis-regulatory element for these processes.

In contrast to “writers”, “erasers” are proteins that remove specific RNA methylation, also known as demethylases, which include fat mass and obesity-associated (FTO) and ALKBH5. FTO is a member of the ALKB family and was the first demethylase discovered. It has been shown to affect the RNA-binding ability of the splicing factor SRSF2, thus regulates pre-mRNA splicing^29–32^. In vitro studies incubating wild-type and mutant FTO proteins with methylated substrates showed that FTO proteins exert demethylating activity on m^6^A in single-stranded RNA^33^. ALKBH5 is another member of the ALKB family that found to have demethylation activity^34^. ALKBH5 colocalizes with nuclear speckles in an RNase A-sensitive manner and can directly catalyze the demethylation of m^6^A-methylated adenosine, which is in contrast to the oxidation reaction catalyzed by FTO^35^. Demethylation by ALKBH5 also affects the efficiency of nascent mRNA synthesis and splicing^36^.

m^6^A is recognized by “readers”. “Readers” are a large class of proteins or domains, which can specifically identify different types of RNA methylation and link RNA methylation modification to specific biological function^37^. At present, known m^6^A-binding proteins are YT521-B homology (YTH) domain proteins, including YT521-B homology domain family 1 (YTHDF1), YTHDF2, YTHDF3, YT521-B homology domain containing 1 (YTHDC1), YTHDC2, and the heterogeneous nuclear ribonucleoprotein (HNRNP) family proteins HNRNPA2B1 and HNRNPC^38^. m^6^A mRNA modifications function in primarily two ways: modulation of the structure of the methylated transcript to prevent or induce protein-RNA interactions and direct recognition by m^6^A-binding proteins, which induces subsequent reactions^25^. A class of proteins containing YTH functional domains has been shown to bind to m^6^A^39^. Among these, YTHDF1, YTHDF2, YTHDF3, YTHDC1, and YTHDC2 have been confirmed to be m^6^A-binding proteins^39^. YTHDF1 primarily affects the gene translations by m^6^A modification, whereas YTHDF2 primarily affects their degradation, and YTHDC1 affects splicing. On the other hand, HNRNPC is an abundant nuclear RNA-binding protein that is involved in pre-mRNA processing^40^. Study has shown that HNRNPC regulates the abundance and alternative splicing of target transcripts via binding to m^6^A and RNA^41^. Dynamic and reversible mRNA m^6^A modification is shown in Fig. 2.

## Epigenetic regulations in ischemia–reperfusion injury

**Fig. 3:**
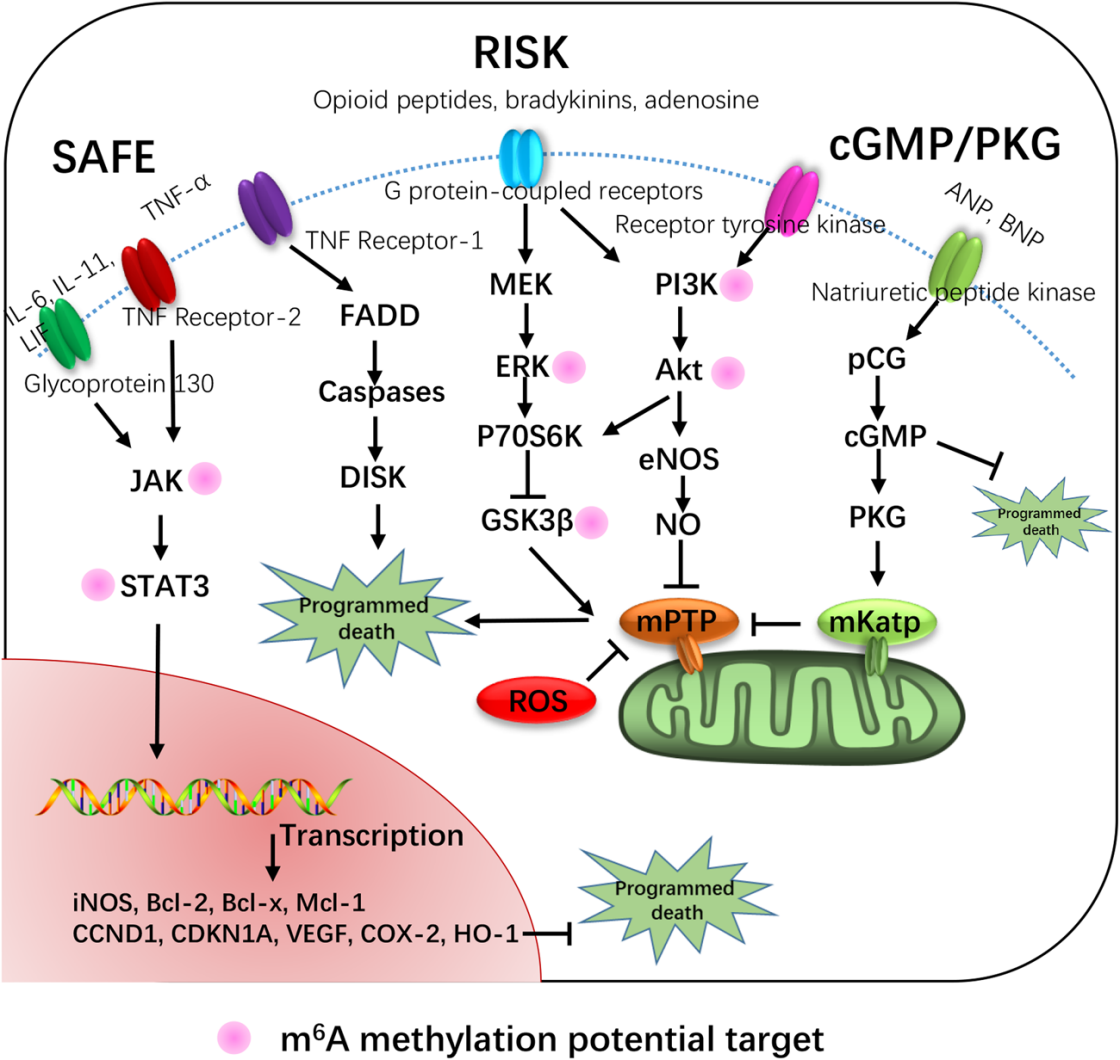
Overview of the target cell signaling pathways associated with I/R injury. The RISK pathway is activated by opioid peptides, bradykinins, adenosine, receptor tyrosine kinase G protein-coupled receptors, erythropoietin, cytokines, insulin, or insulin-like growth factor-1. The cGMP/PKG pathway is activated by external stimuli (ANP and BNP) via the activation of natriuretic peptide receptors A and B. The SAFE pathway is activated by IL-6, IL-11, leukemia inhibitory factor (LIF), or TNF-α via the glycoprotein 130 receptor or TNF-α type 2 receptor. Among these pathways, the potential m^6^A methylation targets have been labeled with pink circle.

I/R injury occurs in various tissues and organs, including the heart^42^, liver^43–45^, kidneys^46^, skin^47^, lungs^48–50^, muscle^51,52^, eye^53,54^, brain^55,56^, blood vessels^57^, and mesentery^58,59^. The pathophysiology of I/R injury in these organs is generally similar^60,61^. I/R injury is primarily characterized by neutrophil infiltration and the burst production of ROS. This excess production of ROS results in oxidative stress in tissues, which leads to cell death and eventually organ dysfunction. The reperfusion injury salvage kinase (RISK)^62–65^, survival activating factor enhancement (SAFE)^66–68^, and cyclic guanosine monophosphate (cGMP)-protein kinase G (cGMP-dependent kinase) signaling pathways^69,70^ are the major pathways involved in the protective effects of various interventions in different organs against I/R injury (Fig. 3). Recently, epigenetic regulation has been suggested to play important roles in I/R injury.

Two major types of epigenetic regulation, histone acetylation and DNA methylation, have been implicated in the pathogenesis of I/R injury. During I/R-induced lung injury, the histone acetylation inhibitor trichostatin A (TSA) has been shown to suppress lung inflammation by inhibiting apoptosis and the phosphorylation of ERK, JNK, and p38^71^. Histone acetylation can also promote the acetylation and release of HMGB1 from hepatocyte nuclei^72^, inhibit MPO activity in lung tissue^73^, and promote the production of antioxidant enzymes, such as FoxO3a and SOD, in cardiac cells^74^, suggesting that histone acetylation can effectively regulate inflammatory infiltration and the oxidative stress response in I/R injury in different tissues and organs. Recent studies on DNA methylation in I/R injury have focused on the kidneys^75,76^, heart^77–79^, and brain^80^, which showed that inhibition of DNA methylation can protect organs from different kinds of I/R injury.

Recently, studies have reported that m^6^A is closely associated with oxidative stress. Li et al.^81^ demonstrated that p21 can induce oxidative stress and regulate cellular senescence through m^6^A and m^5^C RNA modifications. They showed that oxidative stress altered m^6^A levels, thereby affecting mRNA translation^82^. Recently, Xiang Zhong et al.^83^ reported that knockout of the liver circadian clock gene *Bmal1* in mice resulted in abnormal liver lipid metabolism, accompanied with increase of mRNA m^6^A levels and a loss of mRNA m^6^A circadian rhythm. Moreover, in *Bmal1* deletion mice, m^6^A-seq showed elevated m^6^A methylation of *PPAR*α, a key transcription factor that regulates lipid and lipoprotein metabolism in the liver, suggesting that *Bmal1* may regulate hepatic lipid metabolism through m^6^A RNA methylation of *PPAR*α^83^. Inhibition of m^6^A RNA methylation increased the stability and longevity of PPARα mRNA through YTHDF2 by regulating PPARα transcription and translation, reducing lipid accumulation in hepatocytes in vitro^83^. These studies suggest that oxidative stress is involved in m^6^A methylation and that m^6^A may also affect mRNA translation by inducing oxidative stress, indicating that m^6^A and oxidative stress regulate each other. However, whether this interaction exists in I/R injury remains as a question.

Interestingly, m^6^A has been reported in recovery of translational efficiency following hypoxic stress, affecting cell survival^84^. Myocardial infarction-associated transcript (MIAT), a hypoxia-responsive lncRNA, has been identified as a target gene of ALKBH1-related m^6^A mRNA modification. Wu L et al. found that m^6^A mRNA, but not 5-methylcytosine DNA methylation, in leukocyte was reduced in atherosclerosis patients with increased carotid plaque size. They further found that LDL was an independent risk factor in reducing the level of m^6^A and the progression of plaque formation^85^. Mechanistically, they showed that ox-LDL-induced m^6^A demethylation facilitated HIF1α binding to the ALKBH1-demethylated MIAT promoter and activating MIAT transcription^85^. These studies indicate mRNA modifications by m^6^A may take part in hypoxic-related ischemia or I/R disease.

## m^6^A RNA methylation regulates ischemia/reperfusion injury in the heart

Current therapies for ischemic heart disease and adverse post-ischemic cardiac remodeling have limited efficacy. Although the roles of various transcription factors and transcription coactivators have been evaluated, studies on the post-transcriptional regulation of mRNAs that regulate the key proteins and cardiac function are still at the initial stage. Most recently, using whole-genome m^6^A sequencing analysis, Dom et al.^86^ revealed the presence of m^6^A and its dynamic changes in cardiomyocyte hypertrophy. In this study, they found that METTL3 is a key RNA-modifying protein that catalyzes m^6^A methylation of specific mRNA subgroups, which lead to cardiomyocyte hypertrophy. They further showed that enhancement of METTL3 is sufficient to induce cardiomyocyte hypertrophy in the absence of additional stimulation in vitro. Inhibition of METTL3 had no effect on cardiomyocyte hypertrophy under normal serum condition but effectively blocked the development of cardiomyocyte hypertrophy when additional serum was provided^86^. Increase in m^6^A results in the adaptive growth of cardiomyocytes, whereas decrease in m^6^A induces eccentricity and adverse cardiomyocyte geometry. Therefore, alterations in METTL3 level affects m^6^A metabolism and induces spontaneous cardiomyocyte remodeling^86^. This indicates that METTL3-mediated m^6^A modification is sufficient to regulate genes that responsible for cardiac remodeling and function. Thus, METTL3 may be a potential therapeutic target for pathological cardiac remodeling.

Cardiomyocyte death occurs during myocardial I/R and has a critical role in myocardial I/R injury. m^6^A RNA methylation has been shown to closely to the pathogenesis of myocardial I/R injury. Cardiac METTL3 protein level was increased accompanied with decreased myocardial cell viability in mice subjected to myocardial I/R^87^. In line with these findings, in neonatal cardiomyocytes subjected to hypoxia/reoxygenation, upregulation of METTL3 decreased autophagic flux and promoted the cell apoptosis, whereas knockout of METTL3 enhanced the cell viability^87^. These findings highlight the important role of METTL3, or m^6^A modifications in general, in myocardial I/R injury. Indeed, in the same model in cardiomyocytes subjected to hypoxia/reoxygenation, overexpression of RNA demethylase ALKBH5 reversed METTL3-induced cell injury^87^, confirming the role of m^6^A modifications in myocardial I/R injury.

FTO, an m^6^A demethylase that regulates transcriptomic m^6^A modifications in mRNA^88^, is associated with metabolic disorders such as diabetes and obesity, diseases in which the heart is vulnerable to I/R injury. In mammals, cardiac FTO expression is decreased in the failing hearts of human, pig, and mouse, as well as in mouse cardiomyocytes under hypoxia^89^. In cardiomyocytes under hypoxia or mice with heart failure, overexpression of FTO attenuates ischemia-induced cardiac remodeling, increases cardiac contractile protein expression, and improves cardiac contractility^89^, suggesting that FTO would be a therapeutic target for heart failure. Moreover, studies indicated that the cardioprotective mechanism of FTO is mediated by the selective demethylation of transcripts related to cardiac contraction under ischemia, which increases mRNA stability and protein expression^90^.

In addition to its role in I/R injury, m^6^A modifications have been shown to have a role in anesthetic post-conditioning cardioprotection. In H_2_O_2_-induced senescent H9c2 cells, hypoxia/reoxygenation was found to increase the level of m^6^A methylation globally, where post-conditioning with dexmedetomidine, a widely used anesthetic, reduce m^6^A methylation and attenuate cell death^91^. They further showed that dexmedetomidine post-conditioning increases lncRNA H19 by upregulating ALKBH5 and reduces hypoxia/reoxygenation-induced cell death^91^. However, whether the hypoxia/reoxygenation-induced global m^6^A changes apply to genes other than H19 and whether they play roles in dexmedetomidine post-conditioning cellular protective effects are unclear. Thus, exploring the precise mechanism of m^6^A methylation may facilitate the optimization of ischemic cardiac preconditioning or post-conditioning in protecting the heart against I/R injury.

## m^6^A RNA methylation regulates ischemia/reperfusion injury in the brain

The fate of neurons after brain I/R injury is determined by a series of complex biochemical and molecular events, including excitotoxicity^92^, ion imbalance^93^, oxidative stress^94^, endoplasmic reticulum stress^95^, apoptosis^96^, and inflammation^97^. These processes can facilitate rapid changes in the ischemic-sensitive transcriptome. In addition, post-ischemic pathophysiological changes can be fine-tuned by the regulation of post-transcriptional RNA levels^98^ via non-coding RNAs, RNA-binding proteins, and epigenetic post-transcriptional modifications^99,100^. The “eraser” of m^6^A methylation, FTO, is highly enriched in the brain, where its deletion results in impaired dopaminergic neurotransmission and congenital microcephaly^101^. In mammals, m^6^A modifications are the most abundant in the brain and serve to regulate synaptic plasticity, axonal growth, learning and memory, and stress responses^102^. Using the Arraystar mouse epitranscriptome chip, Chokkalla et al.^103^ first demonstrated that m^6^A modifications were increased in 122 mRNAs and 17 lncRNAs and decreased in 15 mRNAs and 3 lncRNAs after 12 hours of transient ischemia and reperfusion in mice. GO/pathway analysis revealed that these mRNAs with altered m^6^A modifications were enriched in biological processes such as inflammation, apoptosis, and transcriptional regulation. Most recently, Diao et al.^104^ showed that, in primary hippocampal neurons, hypoxia/reoxygenation-induced activation of PTEN along with increased cell pyroptosis, which reversed by hypothermia. Interestingly, global m^6^A-methylated RNA, and PTEN methylation in specific, were increased in neurons subjected to hypoxia/reoxygenation, whereas these changes were decreased after hypothermia treatment. Similarly, inhibition of PTEN transcription, the PTEN RNA decay rate was reduced to baseline level after hypothermia treatment^104^, suggesting that hypothermia may confer protective effects through m6A modification of PTEN. Together, these results provide evidence of the involvement of m^6^A modifications in I/R injury in the brain.

## m^6^A RNA methylation regulates ischemia/reperfusion injury in the kidney

During renal I/R injury, excess ROS production results in renal tubular necrosis and renal dysfunction^105,106^. Recent studies have shown that FTO mediates m^6^A mRNA demethylation in the 3′-UTR of peroxisome proliferator-activated receptor γ coactivator-1α (PGC-1α) in renal cells, which increases PGC-1α mRNA stability and protein expression. This increased PGC-1α expression enhanced mitochondrial activity and induced oxidative stress in HEK 293 T renal cells, suggesting that the regulation of m^6^A methylation is closely associated with oxidative stress, the major adverse event of renal I/R injury, in renal cells^107^. During renal I/R injury, acute kidney injury often progresses to chronic kidney injury, and interstitial fibrosis has an important role in this process^108^. Yes-associated protein 1 (YAP1) is involved in kidney regeneration and fibrosis through its proliferation-promoting and pro-fibrosis functions. Interstitial fibrosis and abnormal tubular differentiation are associated with a continuous increase in the activation of YAP1. YAP1 mRNA methylation levels were reduced in HK-2 cells with METTL14 knockdown and in the kidney from METTL14 knockout mice. Loss of METTL14 function resulted in decreased YAP1 methylation levels and increased YAP1 protein translation^109^. Inhibition of the YAP1-TEAD pathway by peptide 17 eliminated the protective effect of METTL14 on renal I/R in vivo and in vitro, indicating that the role of METTL14 in renal I/R is dependent on the activation of YAP1 and the YAP1-TEAD pathway by RNA methylation^109^. This suggests that the regulation of m^6^A RNA methylation is involved in the development and progression of renal I/R injury.

## Summary and outlook

In summary, research on m^6^A modifications has revealed key regulatory enzymes and proteins involved in the process, including methyltransferases, demethylases, and binding proteins. The related regulatory mechanisms, such as microRNAs, are becoming better understood as well. RNA modifications with m^6^A play important regulatory roles in RNA processing and I/R injury (Table 1). Through the combined action of “writers” and “erasers,” the m^6^A levels on RNA can be dynamically regulated, and fine regulation of RNA processing can be achieved through the recruitment of various binding proteins. However, most of the currently known functions of m^6^A have been inferred from the phenotypic consequences of manipulating m^6^A “writers” and “erasers.” The identification of individual mRNA m^6^A sites is still challenging owing to the limitations of current experimental and sequencing technologies. m^6^A modification site mismatches have been achieved at single-base accuracy by modifying DNA polymerases to detect m^6^A transcriptome modification levels^110^. Techniques for the direct detection of RNA modifications on transcripts using nanopore sequencing^111^ have become popular, and these technologies are essential for elucidating the dynamic regulatory mechanisms of m^6^A modification and their molecular functions. The RNA modification mechanisms regulating gene expression during I/R injury will certainly become clearer with additional research and the development of more advanced technologies.

**Table 1:** RNA modifications with m^6^A in RNA processing and I/R injury.

Type of I/R	In vivo or in vitro	Model	Effector	Type of effector	Expression	Cell or tissue	Targeted genes	mRNA stability and protein expression	Physiological effect	Ref.
Myocardial I/R	In vivo	Ischemia 30 min and reperfusion 60 min	METTL3	Writer	Increase	Hear tissue from mouse	TFEB	↓ expression	Leads to heart I/R injury	^87^
	In vitro	Hypoxia 4 h and reoxygenation 3 h	METTL3	Writer	Increase	H9c2 and primary neonatal mouse ventricular cardiomyocytes	TFEB	↓ expression	Enhances apoptosis in H/R-treated cardiomyocytes	
Myocardial I/R	In vivo	Swine: ischemia 120 min and reperfusion 2, 4, 12, 20 w; mouse: ischemia 120 min and reperfusion 4 h, 1 d, 1 w, 4 w;	FTO	Eraser	Decrease	Hear tissue from human, swine and mouse	SERCA2a	↑ expression	Leads to heart I/R injury	^89^
	In vitro	Hypoxia for 16 h	FTO	Eraser	Decrease	Primary cardiac myocyte	SERCA2a	↑ expression	Leads to cardiomyocyte dysfunction	
Brain I/R	In vivo	Ischemia 1 h and reperfusion 3, 6, 12, 24 h	FTO	Eraser	Decrease	Peri-infarct ipsilateral cortical tissue from mouse	No mention	No mention	No functional verification	^103^
Renal I/R	In vivo	Ischemia 30 min and reperfusion 48 h	METTL14	Writer	Increase	Renal tissue from human and mouse	YAP1	↑ stability	Leads to renal I/R injury	^109,112–114^
	In vitro	Hypoxia 12 h and reoxygenation 6,12, 24 h	METTL14	Writer	Increase	HK-2 cell	YAP1	↑ stability	Leads to HK-2 cell I/R injury	
